# Transvaginal repair of a rectovaginal fistula caused by transvaginal mesh prolapse surgery

**DOI:** 10.1002/iju5.12448

**Published:** 2022-04-13

**Authors:** Aika Matsuyama, Kumiko Kato, Hiroki Sai, Akinobu Ishiyama, Takashi Kato, Satoshi Inoue, Hiroki Hirabayashi, Shoji Suzuki

**Affiliations:** ^1^ Department of Urology Japanese Red Cross Aichi Medical Center Nagoya Daiichi Hospital Nagoya Japan; ^2^ Department of Female Urology Japanese Red Cross Aichi Medical Center Nagoya Daiichi Hospital Nagoya Japan

**Keywords:** mesh complication, pelvic organ prolapse, polypropylene mesh, rectovaginal fistula, transvaginal mesh prolapse surgery

## Abstract

**Introduction:**

Transvaginal mesh surgery can cause mesh complications including rare rectovaginal fistula. We report a case of a rectovaginal fistula treated transvaginally without colostomy.

**Case presentation:**

A 57‐year‐old female was referred to us due to post‐hysterectomy prolapse and had transvaginal mesh surgery. She underwent transvaginal hysterectomy because of uterine prolapse at age 33 and had taken steroids to treat pemphigus. Two years later, she developed vaginal bleeding and discharge. Transvaginal mesh removal was planned to treat vaginal mesh exposure, but immediately before the operation digital rectal examination revealed rectovaginal fistula. Mesh removal and fistula closure were performed transvaginally without colostomy. Three years of follow‐up showed no recurrence of mesh exposure, fistula, or prolapse.

**Conclusion:**

Rectovaginal fistula following mesh surgery may be treated transvaginally without colostomy if infection is minimal. To evaluate mesh exposure on the posterior vaginal wall, rectal examination should be done along with vaginal examination.

**Abbreviations & Acronyms**
FDA: U.S. Food and Drug Administration
LSC: laparoscopic sacrocolpopexy
NTR: native tissue repair
POP: pelvic organ prolapse
RASC: robot‐assisted sacrocolpopexy
RVF: rectovaginal fistula
SUI: stress urinary incontinence
TVM: transvaginal mesh surgery

**Keynote message**

RVF following TVM may be treated with a transvaginal approach without colostomy if the infection is minimal. It is important to evaluate both the mesh exposure site and the possibility of fistula meticulously.

## Introduction

Female pelvic floor disorders including POP are highly prevalent conditions. The lifetime risk of American women for either POP or SUI is estimated to be 20.0%: 12.6% for POP and 13.6% for SUI, respectively.^1^ POP surgeries are generally classified into three approaches: NTR, TVM, and LSC. Usage of mesh began in the 1990s and self‐cut TVM was introduced in Japan in 2005.^2^ TVM became popular due to its low invasiveness, uterine preservation, and low recurrence rate, but mesh complication reports gradually increased. The FDA issued safety communications in 2011 on the transvaginal placement of synthetic mesh for POP and SUI. Thereafter, NTR gained attention again. Furthermore, the number of LSC including RASC has been increasing though these are also associated with non‐negligible mesh complications. Among mesh complications, vaginal mesh exposure is the most frequent.^3^ Conversely, RVF is one of the least frequent, and management methods are not well established.^4^, ^5^, ^6^, ^7^, ^8^, ^9^ We report a case of RVF following TVM which was successfully repaired by a transvaginal approach without colostomy.

## Case presentation

A 57‐year‐old parous female was referred to our department due to post‐hysterectomy prolapse and voiding difficulty which worsened over the past 10 years. At the age of 33, she underwent transvaginal hysterectomy and colporrhaphy due to uterine prolapse. Additionally, she had been taking oral steroids (11 mg of prednisolone per day) for over 8 years due to pemphigus. She underwent Elevate^®^‐type TVM using self‐cut polypropylene mesh. The postoperative course was uneventful.

**Fig. 1:**
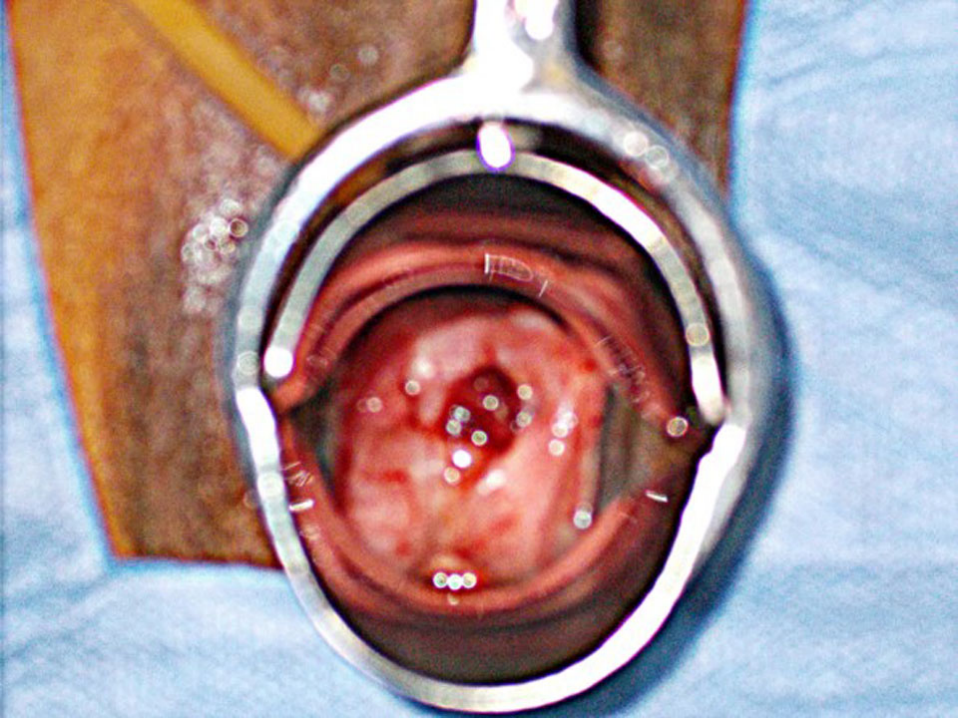
Vaginal examination showed a small mesh exposure (0.8 cm in diameter) in the posterior vaginal wall near the vaginal stump (low quality, but only available image). [Colour figure can be viewed at wileyonlinelibrary.com]

**Fig. 2:**
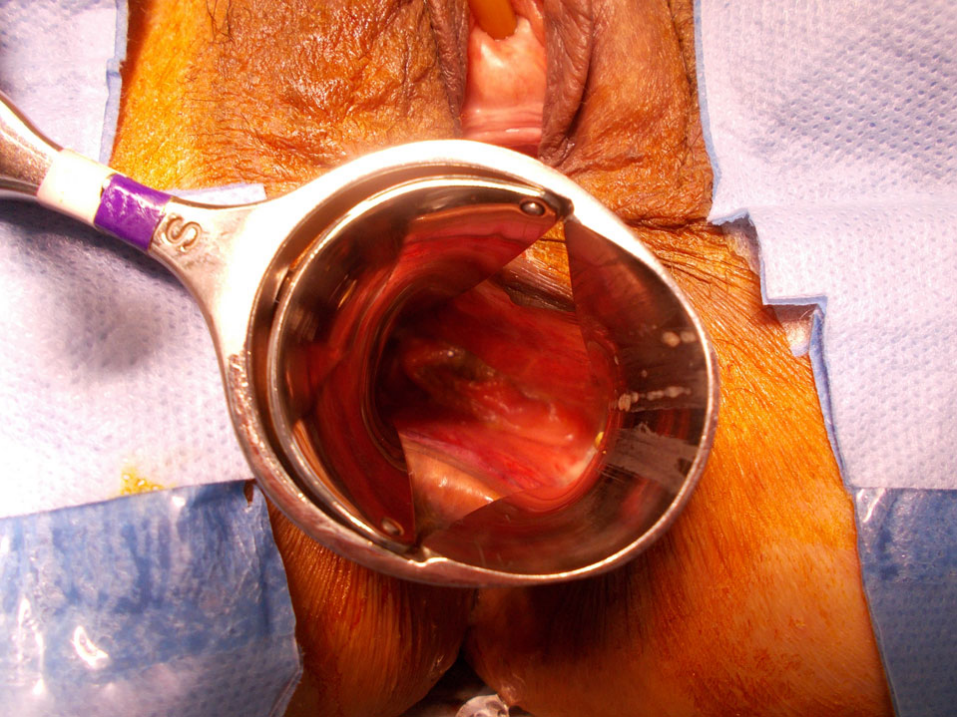
Rectal examination revealed a small mesh exposure in the anterior rectal wall; thus, a RVF was diagnosed in the operating room. [Colour figure can be viewed at wileyonlinelibrary.com]

**Fig. 3:**
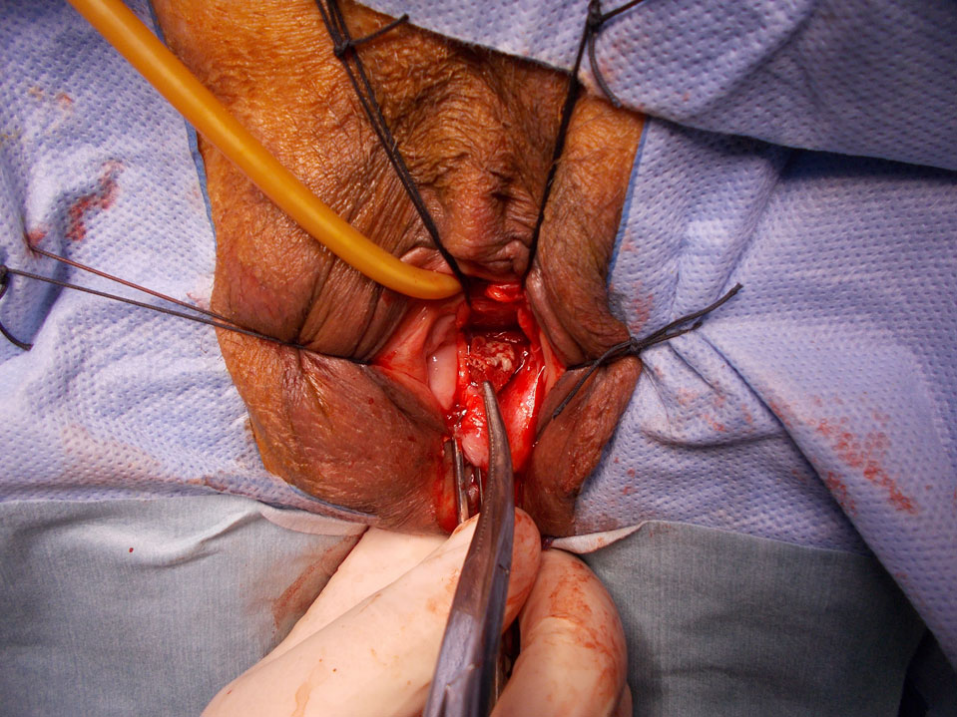
Following fluid dissection using epinephrine solution, a midline incision was made on the posterior vaginal wall. After identifying the mesh edge, we applied traction to the mesh using clamps and threads, and dissected the mesh gradually from the surrounding tissue. After semi‐total removal of the mesh (excluding the arms' distal parts), we found a small hole (approximately 1 cm in diameter) between the vaginal and rectal walls, which was closed with 3 layers of 3–0 absorbable sutures. Suspending the anterior rectal wall with a surgeon's finger placed in the rectum helped suture adequately. [Colour figure can be viewed at wileyonlinelibrary.com]

**Fig. 4:**
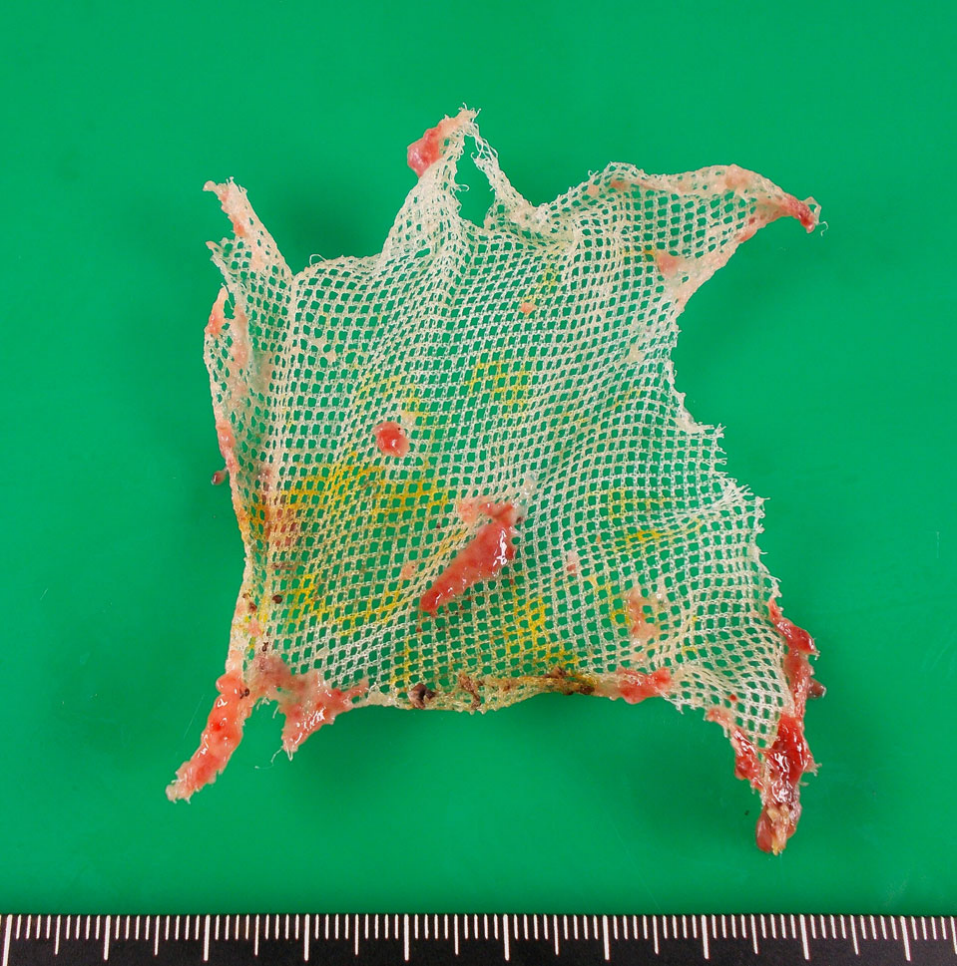
Semi‐totally removed mesh excluding the arms' distal parts. In previous TVM, Polyform^®^ (Boston Scientific, Marlborough, MA, USA) was cut into the shape of Elevate^®^ (two anterior transobturator mesh arms and two posterior sacrospinous mesh arms) following a paper pattern. [Colour figure can be viewed at wileyonlinelibrary.com]

Two years later, however, she complained of vaginal bleeding and a brownish discharge. She visited a gynecologist and a polyp near the vaginal stump was observed (biopsy: granulation tissue). She was referred to our hospital again, and a small mesh exposure (0.8 cm in diameter) at the posterior wall was diagnosed during the vaginal examination (Fig. 1). Transvaginal mesh removal under spinal anesthesia was planned. Immediately before the operation digital rectal examination revealed rectal mesh exposure and RVF (Fig. 2). According to the International Urogynecological Association/International Continence Society classification system categorizing prosthesis/graft complication,^10^, ^11^ this case was 5BT4S3. Following transvaginal mesh removal (semi‐total removal excluding the arms' distal parts), fistula closure could be securely completed with three layers of absorbable sutures (details in Figs 3 and 4). Exposure of the left posterior mesh arm was the suspected cause of the fistula. Postoperatively, she had nothing by mouth for 6 days. No recurrences of mesh exposure, fistula, or prolapse have been found in the 3 years of follow‐up.

## Discussion

RVF is defined as a pathological epithelialized communication between the posterior vaginal wall and the anterior rectal wall. In general, 85% of RVF are caused by obstetric trauma, and 5–7% of RVF is attributed to pelvic and rectal surgery while inflammatory bowel disease, malignancy, and radiotherapy cover most of the remaining etiologies.^12^ With the spread of mesh prolapse surgery, postoperative RVF cases have been reported over the last two decades.^4^, ^5^, ^6^, ^7^, ^8^, ^9^

Mesh complications are more commonly reported after TVM, but abdominal sacrocolpopexy, LSC, and RASC are also causes. Regarding TVM, vaginal mesh exposure is the most frequent complication and its reported prevalence is 2.7–17%^3^ though the prevalence is low in Japan (0.7–3.2%) likely due to surgical education.^2^, ^13^ Conversely, RVF formation is rare at a reported prevalence of 0.15%.^4^ Dwyer *et al*. and Hilger *et al*. reported a case who developed RVF 5 days and 3 months after TVM, respectively.^5^, ^6^ Ouaissi *et al*. reported a series of RVF in 5 patients which occurred on average 18 months after mesh prolapse surgery (transabdominal 3, TVM 2).^7^ Choi *et al*. reported a series of RVF in 10 patients with an average onset of 7.1 months after POP repair (TVM 8, LSC 1, RASC 1).^8^ In Japan, Koide *et al*. reported an RVF which was found due to hematochezia one month following TVM.^9^ RVF symptoms include hematochezia, the passage of air and stool from the vagina, vaginal bleeding and discharge, and dyspareunia. As our case showed only vaginal bleeding and discharge, we diagnosed vaginal exposure but overlooked rectal exposure and RVF at the outpatient clinic.

Regarding mesh complications, multiple risk factors have been indicated; history of previous POP surgery, radiation, low estrogen, smoking, diabetes mellitus, steroid usage, and inflammatory bowel diseases.^14^ Our case had a history of POP repair and a long‐term steroid usage. Concomitant hysterectomy and SUI surgery are known to increase mesh exposure.^14^, ^15^, ^16^, ^17^, ^18^ Furthermore, surgeons' experience levels influence the risk of complications related to improper vaginal dissection layer,^2^, ^13^ and accidental punctures of vaginal, vesical or rectal walls. Kato *et al*. reported that experienced surgeons (TVM > 50 cases) had significantly less prevalence of vaginal mesh exposure, bladder injury and blood transfusion.^2^ Postoperative mesh infection and hematoma are also associated with a higher complication rate.

Various surgical approaches have been attempted to treat RVF depending on its size, location, etiology, the state of the surrounding tissue, infection, and prior repair attempts. For small (<2 cm), non‐recurrent, low‐type RVF without significant infection, transvaginal, transrectal, or transperitoneal closure is preferable due to its low invasiveness.^12^, ^19^, ^20^ Transabdominal repair with or without bowel diversion is mainly used when the RVF is large, high‐type, recurrent, or complex (accompanied by infection, caused by inflammatory bowel disease, malignancy, or radiotherapy).^12^, ^19^, ^20^ Additionally, the Martius flap procedure and the Gracilis muscle interposition are used in difficult or recurrent cases.^5^, ^19^, ^20^

Regarding RVF following mesh prolapse surgery, Choi *et al*. reported that a mean of 4.4 surgeries was needed, with 40% requiring a colostomy.^8^ Some authors have recommended that removal of enough mesh and control of infection are mandatory.^7^, ^8^ In our case, RVF was small and found 2 years after TVM; the mesh adhered to the surrounding tissue and the infection around the mesh was minimal. Although the fistula was located near the vaginal stump, it could be well elevated with a surgeon's finger in the rectum; thus, the mesh could be sub‐totally removed, the vaginal and rectal walls could be separated and mobilized adequately, and the fistula between these walls could be securely sutured without tension. In similar situations, transvaginal simple closure has a high probability of success. The repair approach should be decided on a patient‐by‐patient basis, consulting gastrointestinal surgeons if necessary.

To conclude, precise assessment of the site and size of mesh exposure as well as the possible presence of fistula or infection is important. We recommend not only vaginal examination but also rectal examination when mesh exposure of the posterior vaginal wall is suspected.

## Conflict of interest

The authors declare no conflict of interest.

## Author Contributions

Aika Matsuyama: Conceptualization; data curation; formal analysis; writing – original draft. Kumiko Kato: Conceptualization; data curation; formal analysis; writing – review and editing. Hiroki Sai: Data curation. Akinobu Ishiyama: Data curation. Takashi Kato: Data curation. Satoshi Inoue: Data curation. Hiroki Hirabayashi: Supervision. Shoji Suzuki: Supervision.

## Approval of the research protocol by an Institutional Reviewer Board

Not applicable.

## Informed consent

Written informed consent was obtained from the patient.

## Registry and the Registration No. of the study/trial

Not applicable.
